# Long Noncoding RNA 00472: A Novel Biomarker in Human Diseases

**DOI:** 10.3389/fphar.2021.726908

**Published:** 2021-12-20

**Authors:** Dan-yang Ren, Xin-rong Yuan, Cai-xia Tu, Jian-ling Shen, Yun-wei Li, Ai-hua Yan, Yi Ru, Hui-yun Han, Yan-ming Yang, Yan Liu, Hui-ying Li

**Affiliations:** ^1^ Pharmaceutical Preparation Section, Children’s Hospital of Kunming Medical University, Kunming, China; ^2^ Department of Neurology, Xiangya Hospital, Central South University, Changsha, China

**Keywords:** biological functions, clinical application, LINC00472, mechanism, biomarker

## Abstract

Long non-coding RNAs (lncRNAs) play important roles in human diseases. They control gene expression levels and influence various biological processes through multiple mechanisms. Functional abnormalities in lncRNAs are strongly associated with occurrence and development of various diseases. LINC00472, which is located on chromosome 6q13, is involved in several human diseases, particularly cancers of the breast, lung, liver, osteosarcoma, bladder, colorectal, ovarian, pancreatic and stomach. Importantly, LINC00472 can be used as a biomarker for breast cancer cell sensitivity to chemotherapeutic regimens, including doxorubicin. LINC00472 is regulated by microRNAs and several signaling pathways. However, the significance of LINC00472 in human diseases has not been clearly established. In this review, we elucidate on the significance of LINC00472 in various human diseases, indicating that LINC00472 may be a diagnostic, prognostic as well as therapeutic target for these diseases.

## Introduction

Based on their sequence length, there are two types of noncoding RNAs (ncRNAs): long non-coding RNAs (LncRNAs) and small ncRNAs (SncRNAs) (Tang et al., 2020; El-Ashmawy et al., 2021). The former is a member of a large family of ncRNAs, which have a length of >200 nucleotides but, it does not code for proteins (Cantile et al., 2021; Qi et al., 2021). LncRNAs play several functions in human diseases, such as cell proliferation, growth, cell cycle, differentiation, migration, apoptosis, invasiveness as well as drug resistance (Cantile et al., 2021; Zheng et al., 2021). Importantly, lncRNAs are closely associated with disease diagnoses, prognoses, and therapeutic outcomes (Li et al., 2021a).

**FIGURE 1:**
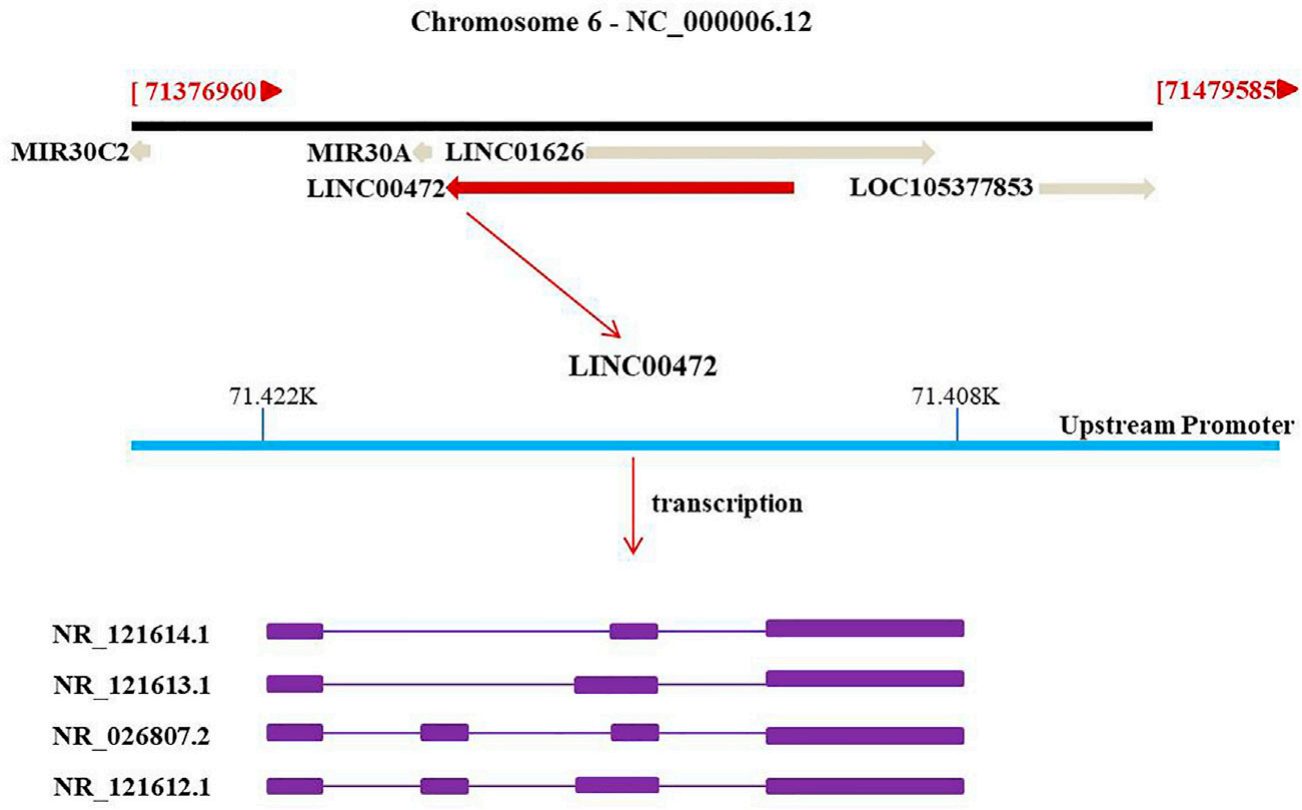
Schematic diagram of the formation of LINC00472.

The NCBI database (https://www.ncbi.nlm.nih.gov/) revealed that the LINC00472 gene is located on chromosome 6q13 antisense chain. This gene sequence can be transcribed into four different transcripts: NR_121,614.1, NR_121,613.1, NR_026,807.2 and NR_121,612.1 (Figure 1). LINC00472 is a novel LncRNAs that is closely associated with tumorigenesis and tumor progression in various cancers including breast cancer (BC) (Shen et al., 2015a; Lu et al., 2018; Wang et al., 2019a; Li et al., 2021a; Shao et al., 2021), lung cancer (LC) (Su et al., 2018; Mao et al., 2019; Zou et al., 2019; Deng et al., 2020), hepatocellular carcinoma (HCC) (Chen et al., 2019), osteosarcoma (OS) (Zhang et al., 2020), colorectal cancer (CRC) (Ye et al., 2018), epithelial ovarian cancer (OC) (Fu et al., 2016), pancreatic cancer (PC) (Bi and Wang, 2021), and gastric cancer (GC) (Tsai et al., 2019). As a tumor suppressor, LINC00472 suppresses cell proliferation, migration as well as invasion. Moreover, it enhances apoptosis. The regulatory roles of LINC00472 in non-cancerous diseases, such as atrial fibrillation (AF) (Wang et al., 2019b), osteoporosis (OP) (Guo et al., 2020), atherosclerosis (Jing et al., 2021), sepsis-induced acute hepatic injury (AHI) (Li et al., 2020a), and primary biliary cholangitis (PBC) (Dong et al., 2021), have also been documented. These studies found that LINC00472 is expressed in various diseases and that it affects miRs or signaling through various pathways. This review summarizes the potential regulatory roles of LINC00472 in various diseases, with a focus on its aberrant expressions, related clinical features, potential molecular mechanisms of action, and prospects in clinical applications. Moreover, we predicted the potential future research directions for LINC00472.

### Function of LINC00472 in Human Diseases

**FIGURE 2:**
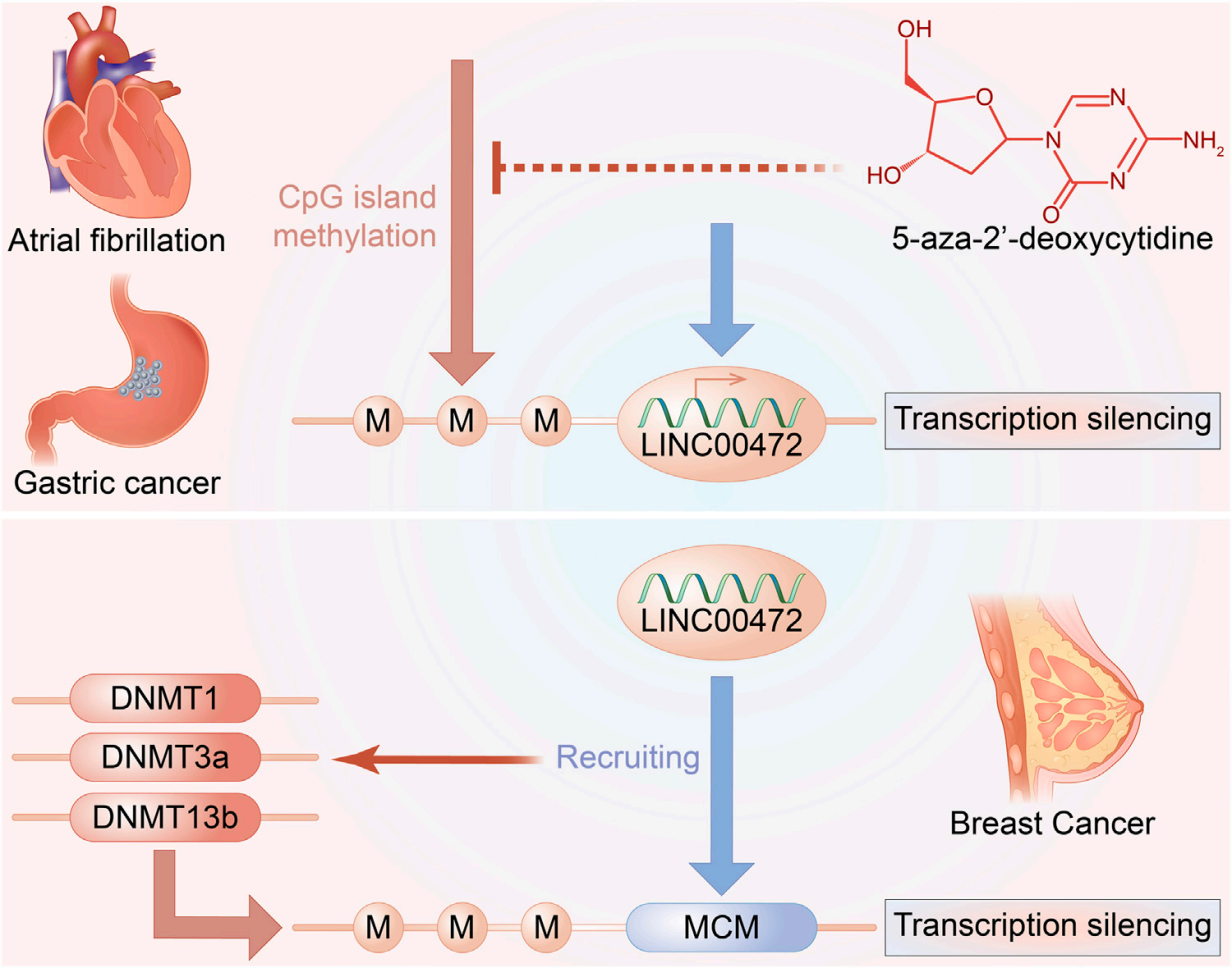
LINC00472 and DNA methylation.

Since 2015, studies on the significance of LINC00472 in various diseases have been increasing. LINC00472 dysregulation has recently been associated with several human diseases, including BC, LC, HCC, OS, CRC, OC, PC, GC, atherosclerosis, AF, OP, sepsis-induced AHI and PBC. In addition, the relationship between LINC00472 expression and various diseases has been documented. These studies elucidated on the clinical characteristics (Table 1), expression levels, and functions (Table 2; Figure 2) of LINC00472.

**TABLE 1:** Expression and clinical characteristics of LINC00472 in different diseases.

**Disease type**	**Expression**	**Number (patients)**	**Cancer cell line**	**Clinical characteristics**	**References**
breast cancer	downregulated	430	MCF7, SKBR3, MDA-MB-231, MDA-MB-453, T47D, Hs578T, ZR-75-1, HCC-1937 and MDA-MB-468	Disease stage, tumor grade, disease-free, overall survival, and lymph node metastasis	Shen et al. (2015a); Lu et al. (2018); Wang et al. (2019a); Li et al. (2021a); Shao et al. (2021)
lung cancer	downregulated	24	A549, H1299, H460, H446, PC-9, H1975, 95D, Calu-3,SPC-A1,H838, H157, H358	TNM clinical stages	Su et al. (2018); Mao et al. (2019); Zou et al. (2019); Deng et al. (2020)
hepatocellular carcinoma	downregulated	109	Huh-7, SMMC-7721	overall survival	Chen et al. (2019)
osteosarcoma	downregulated	49	U2OS and MG63	-	Zhang et al. (2020)
colorectal cancer	downregulated	92	SW480,SW620, HT-29 and HCT-116	-	Ye et al. (2018)
ovarian cancer	downregulated	266	-	Disease stage and tumor grade	Fu et al. (2016)
pancreatic cancer	downregulated	70	SW 1990, BXPC3, Capan-2, and PANC-1	patient’s TNM stage, lymph node metastasis, and overall survival	Bi and Wang, (2021)
gastric cancer	downregulated	42	AGS, AZ-521, HR, TSGH, SNU-1, and NCI-N87	-	Tsai et al. (2019)
atrial fibrillation	downregulated	125	HCM and H9C2 cells	-	Wang et al. (2019b)
osteoporosis	downregulated	55	mice bone marrow mesenchymal stem cells	-	Guo et al. (2020)
atherosclerosis	upregulated	20 samples from normal coronary artery and ath-erosclerotic coronary tissues	vascular smooth muscle cells (VSMCs)	-	Jing et al. (2021)
sepsis-induced acute hepatic injury	upregulated	-	Human liver THLE-3 cells	-	Li et al. (2020a)
primary biliary cholangitis	upregulated	80	HiBECs	Disease stage, and C-IV level	Dong et al. (2021)

**TABLE 2:** Function and mechanism of LINC00472 in different diseases.

**Disease type**	**Expression**	**Role**	**Biological significance**	**Pathway, axis**	**References**
breast cancer	downregulated	Tumor suppressor	cell proliferation, migration, invasion, apoptosis, and cell cycle	miR-141/PDCD4, NF-κB signaling pathway, MEK/ERK signaling pathway, MCM6	Shen et al. (2015a); Lu et al. (2018); Wang et al. (2019a); Li et al. (2021a); Shao et al. (2021)
lung cancer	downregulated	Tumor suppressor	growth	miR-24-3p/DEDD, YBX1, miR-196b-5p, miR-149-3p, miR-4270, p53 signaling pathway, KLLN	Su et al. (2018); Mao et al. (2019); Zou et al. (2019); Deng et al. (2020)
hepatocellular carcinoma	downregulated	Tumor suppressor	cell proliferation, migration, invasion, and apoptosis	miR-93-5p/PDCD4	Chen et al. (2019)
osteosarcoma	downregulated	Tumor suppressor	cell proliferation, colony formation abilities. apoptosis, migration, and invasion	miR-300/FOXO1	Zhang et al. (2020)
colorectal cancer	downregulated	Tumor suppressor	cell proliferation, and apoptosis	miR-196a/PDCD4	Ye et al. (2018)
ovarian cancer	downregulated	-	-	-	Fu et al. (2016)
pancreatic cancer	downregulated	Tumor suppresso	cell proliferation, and apoptosis	miR-23a-3p/FOXO3/BID	Bi and Wang, (2021)
gastric cancer	downregulated	Tumor suppressor	cell growth, and motility	-	Tsai et al. (2019)
atrial fibrillation	downregulated	-	-	-	Wang et al. (2019b)
osteoporosis	downregulated	-	osteogenic differentiation	miR-300/FGFR2	Guo et al. (2020)
atherosclerosis	upregulated	-	cell migration, and proliferation	TNF-α, PDGF-BB, miR-149-3p	Jing et al. (2021)
sepsis-induced acute hepatic injury	upregulated	-	cell viability, and apoptosis	miR-373-3p/TRIM8	Li et al. (2020a)
primary biliary cholangitis	upregulated	-	-	-	Dong et al. (2021)

### LINC00472 in Different Solid Tumor Types

#### Breast Cancer

Globally, breast cancer (BC) is the most prevalent tumor type among women. Its clinical incidences has been steadily increasing over the last 3 decades (Gote et al., 2021; Sharma, 2021). Despite standard chemotherapy and hormonal therapy, recurrence and metastasis account for 90% of mortality in BC patients (Chen et al., 2021a). Therefore, new types of molecular markers are being actively evaluated in the diagnosis and prognosis of BC. In the past 100 years, various murine models have been developed for cancer research (Yang, 2021). For instance, in the past 50 years, human x mouse xenografts, in particular, have been widely used (Yang, 2021). Subcutaneous tumor xenograft models are the commonly used animal models for LncRNAs research in cancers. These models are mainly established by injecting tumor cells into naked mice. In BC, enforced expression of LINC00472 inhibited tumor growth (Lu et al., 2018). Furthermore, tumors from the LINC00472 overexpressing BC cells grow slowly, relative to those without LINC00472 overexpression (Wang et al., 2019a). Importantly, tissue analysis showed that LINC00472 overexpressing BC cells were found to exhibit suppressed malignant abilities (Wang et al., 2019a). Bioinformatics analyses showed that expressions of LINC00472 in BC are regulated by promoter methylation (Shen et al., 2015b). Moreover, LINC00472 induces site-specific DNA methylation and suppresses MCM6 expression by recruiting DNA methyltransferases into MCM6 promoters (Shao et al., 2021).

#### Lung Cancer

Lung cancer (LC), which is the most prominent cause of tumor-associated mortality (Xie et al., 2021), has a global incidence of approximately 1,760,000 cases per year (Su et al., 2021). LC is classified into two types: including small cell LC and non-small cell LC (NSCLC) (Ma et al., 2021a). Clinically, NSCLC accounts for nearly 80–85% of all LC cases (Ma et al., 2021a). Furthermore, lung adenocarcinoma (LUAD), the most prevalent and aggressive histological subtype of LC, with approximately 70% LUAD cases being diagnosed at advanced or metastatic stages (Liu et al., 2021a; Ma et al., 2021a). Even though concurrent radiotherapy (RT) and chemotherapy are recommended as standard treatment options, these approaches significantly limits treatment options (Liu et al., 2021a). To understand the potential cancer-associated molecular mechanisms in LC, studies on antitumor therapies and prognosis are required. In LUAD patients, LINC00472 was of lower expression levels in tumorous tissues than in normal tissues (Su et al., 2018). Moreover, LINC00472 levels in 119 human NSCLC tissues were markedly suppressed, relative to neighboring normal tissues (Zou et al., 2019). Inhibitory roles of overexpressed LINC00472 on lung tumor cell proliferations have been validated *in vivo* (Lu et al., 2018). Studies on NSCLC have reached the same conclusions. *In vivo*, after injecting overexpressed (oe) negative control (NC) or oe-LINC00472, tumor sizes were measured in nude mice (Zou et al., 2019). It was established that LINC00472 expression suppresses NSCLC tumor growth. Further research has confirmed that EMT plays important roles in lung cancer (Zou et al., 2019). In NSCLC, compared to oe-NC group, expression levels of p21, E-cadherin, KLLN, and p53 were markedly elevated. However, those of N-cadherin, Vimentin, as well as cyclin D1 were markedly suppressed in the oe-LINC00472 group (Zou et al., 2019). These findings imply that high LINC00472 levels suppress NSCLC cell development *in vivo*.

#### Hepatocellular Carcinoma

Globally, hepatocellular carcinoma (HCC) is the most prevalent liver cancer type, and the third most important cause of tumor-associated mortality (Shi et al., 2021a; Dai et al., 2021; Hu et al., 2021). However, as a result of local recurrence and metastasis following therapy, most HCC patients exhibit limited benefits and poor outcomes (Hu et al., 2021). Thus, it is vital to evaluate the pathomechanisms that contribute to HCC progression and to establish prognostic biomarkers and treatment targets. As previously reported, in HCC tissues, LINC00472 levels are decreased, relative to those of the nearby normal tissues (Chen et al., 2019). Notably, LINC00472 levels were downregulated in metastatic HCC tissues, relative to non-metastatic tissues (Chen et al., 2019).

#### Colorectal Cancer

Colorectal cancer (CRC), a globally prevalent malignancy, has a growing prevalence, particularly in young adults (Zhang et al., 2021a; Kelm et al., 2021). According to recent statistics, CRC is the second most prevalent cause of tumor-associated deaths, after lung cancer (Ma et al., 2021b). These poor outcomes are attributed to inadequate CRC screening and lack of clinical markers for CRC progression and prognosis (Shi et al., 2021b). Therefore, there is a need to develop innovative CRC therapy techniques (Zhang et al., 2021b). LINC00472 was discovered to be under-expressed in CRC tissues in a recent study (Ye et al., 2018). Nevertheless, Ye et al. (2018) found no substantive association between LINC00472 levels and overall survival in CRC. Ectopic expressions of LINC00472 inhibited CRC tumor proliferation in nude mice (Ye et al., 2018). LINC00472 transfected HT-29 as well as HCT-116 cells exhibited significantly elevated LINC00472 levels, relative to empty vectors (Ye et al., 2018).

#### Ovarian Cancer

Among women, ovarian cancer (OC) is the fifth leading cause of tumor-associated deaths and is correlated with the highest mortality rate among gynecological malignancies worldwide (Wang et al., 2021a; Seborova et al., 2021). Surgery and postoperative platinum-based chemotherapy are the major treatments of OC, according to guidelines of the National Comprehensive Cancer Network (NCCN) (Xu et al., 2021a). Even though surgery and chemotherapy can increase survival, the 5-year survival rate of OC patients remains extremely low (Liu et al., 2021b). Therefore, searching for more sensitive biomarkers, and identifying new therapeutic targets have emerged as OC research trends (Liu et al., 2021b; Xu et al., 2021b). It is hypothesized that LINC00472 expression in epithelial OC may be linked to earlier disease stage and lower tumor grade in recent studies (Fu et al., 2016). However, no discernible variations in LINC00472 expression were found between non-serous and serous carcinomas (Fu et al., 2016). In addition, LINC00472 levels were not correlated with either overall or progression-free survival in OC (Fu et al., 2016).

#### Gastric Cancer

Globally, gastric cancer (GC), the third leading cause of tumor-related mortalities and the fifth most prevalent carcinoma, had an approximated 1,089,103 new morbidities and 768,793 mortalities reported in 2020 (Wang et al., 2021b; Lee et al., 2021). Even though there are many treatment options for GC, prognostic outcomes for late stage GC are poor (Wang et al., 2021b). Thus, it is essential to establish the pathomechanisms involved in the occurrence and development of GC, to develop novel therapeutic approaches (Yang et al., 2021a). When compared to nearby normal tissues, LINC00472 was suppressed in GC tissue samples (Tsai et al., 2019). Moreover, Tumor-specific hyper-methylations of CpG regions upstream of LINC00472 were frequently identified in GC tissues, suggesting that its expression in GC tissues may be reduced (Tsai et al., 2019). The findings demonstrate that the LINC00472 gene can be epigenetically regulated in GC via DNA methylation.

#### Pancreatic Cancer

Pancreatic cancer (PC) is highly common in the digestive tract. It is projected that by 2030, globally, PC will be the second leading cause of tumor-related mortalities with a 5% 5-year survival rate (Li et al., 2021b; Cheng et al., 2021; Xu et al., 2021c; Long et al., 2021). Its prevalence and mortality rates are annually increasing, which makes it an important human health concern (Long et al., 2021). Although surgical resection, comprehensive targeted treatments as well as neoadjuvant therapies are good therapeutic options, early metastasis and invasion, coupled with a lack of effective and specific targeted treatment options are associated with poor prognostic outcomes for PC patients (Liu et al., 2021c). Thus, it is vital to evaluate the mechanisms of PC progression and to establish novel treatment targets for improving survival outcomes of PC patients. In a previous study, RT-qPCR analysis revealed that LINC00472 levels in pancreatic cancer tissues were suppressed, relative to adjacent normal tissues (Bi and Wang, 2021). Moreover, the study identified correlations between LINC00472 low expressions and TNM stages, lymph node metastases and overall survival outcomes but not with gender and age (Bi and Wang, 2021). A stable LINC00472 overexpressing PANC-1 cell line was established and subcutaneously inoculated into nude mice models to validate the *in vivo* effects of LINC00472. Up-regulated LINC00472 levels in nude mice injected with oe-LINC00472–treated cells were confirmed. Tumor growth curves, tumor images as well as weights showed that overexpressed LINC00472 inhibits *in vivo* tumorigenesis (Bi and Wang, 2021). Moreover, overexpressed LINC00472 suppresses Ki-67 levels. Meanwhile, TUNEL staining confirmed that overexpressed LINC00472 induces cell apoptosis (Bi and Wang, 2021). The stable cell line (BXPC3) transfected with lentivirus expressing small hairpin RNA sh-LINC00472, was subcutaneously inoculated into nude mice models to induce tumorigenesis. LINC00472 knockdown in nude mice was ascertained by RT-qPCR (Bi and Wang, 2021). LINC00472 knockdown enhanced pancreatic cancer cell tumorigenicity *in vivo*, suppressed Ki-67 levels as well as cell apoptosis (Bi and Wang, 2021). These results show that LINC00472 inhibits pancreatic cancer development *in vivo*.

#### Osteosarcoma

Osteosarcoma (OS), the most prevalent bone cancer type, is a life-threatening disease, particularly in children and in adolescents below 20 years in age (Gao et al., 2021). It accounts for about 5% of pediatric cancers (Liu et al., 2021d). The diagnosis of OS is primarily based on imaging, and optimal treatment options for OS are surgical resection combined with adjuvant chemotherapy (Liu et al., 2021d). However, survival rates for metastatic or recurrent OS are unfavorable (Yao et al., 2021). Currently, molecular mechanisms involved in OS occurrence and development are unknown, which hinders the development of effective treatment strategies (Liu et al., 2021e). It has been reported that LINC00472 levels were down-regulated in OS patients tissues (Zhang et al., 2020). In OS MG63 cell nude mice xenograft models, tumor growth was found to be decreased in mice injected with sh-LINC00472 transfected cells (Zhang et al., 2020).

### LINC00472 in Non-cancer Diseases

#### Atherosclerosis

Atherosclerosis, which is a maladaptive coronary disease, clinically manifests by arterial hardening as well as narrowing as a result of plaque build-up (Pillai et al., 2021), with early events occurring in the endothelium, which forms the inner surface of the vascular wall (Moreau et al., 2021). Globally, atherosclerosis is a leading cause of myocardial infarction. In 2019, its prevalence in adults aged 60 years and above in European and American countries was 60% (Yang et al., 2021b). Although their roles in atherogenesis have been extensively characterized, the molecular mechanisms associated with altered gene expressions are unknown (Moreau et al., 2021). LINC00472 has also been studied in atherosclerotic coronary tissue, where it was found to be highly expressed (Jing et al., 2021). In VSMCs, LINC00472 promoted cell proliferation and induced cell migration after pcDNA-LINC00472 treatment (Jing et al., 2021). These findings, however, contradict what has been reported in tumors.

#### Osteoporosis

Osteoporosis (OP) is a common bone disease among the elderly. It is characterized by decreased bone density, bone microstructural damage and increased bone fragility (Zhu et al., 2021a). This condition limits patients’ activity and decreases their quality of life, particularly in elderly and postmenopausal women (Jia et al., 2021). The steady state of bones was mainly maintained by osteoblasts and osteoclasts. Abnormal differentiation of osteoblasts and osteoclasts are associated with the loss of bone mass and bone structure strengths, leding to OP development (Ren et al., 2021a; Li et al., 2021c). Therefore, it is important to develop novel therapeutic strategies that can promote osteoblast differentiation while enhancing bone formation. LINC00472 levels have been reported to be markedly lower in osteoporosis patients as compared to healthy control groups (Guo et al., 2020). Guo et al. used ovariectomized (OVX) mice as osteoporosis models and found that expressions of LINC00472 in the OVX group were markedly low, relative to the sham group (Guo et al., 2020). Furthermore, the content of β-CTX (bone metabolism related gene) and activity of ALP (osteogenic differentiation related gene) in serum of mice in OVX group were significantly increased (Guo et al., 2020), indicating that LINC00472 can affect the process of osteogenic differentiation.

#### Atrial Fibrillation

Globally, atrial fibrillation (AF) is the most prevalent heart rhythm disease. It is especially common among the elderly, who account for approximately 0.5% of the global population (Lu et al., 2019). AF is strongly correlated with increased risks of various outcomes, including stroke, heart failure, as well as cardiovascular-related mortality (Ruan et al., 2020). Current AF therapies have various limitations, including high recurrence rates, low tolerance, and possible adverse effects (Du et al., 2020). Furthermore, the underlying pathophysiology and mechanisms of AF remain unknown. As a result, there is an urgent need to evaluate the pathomechanisms underlying AF to aid in development of new effective therapeutic approaches. LINC00472 expression was shown to be low in both cardiac muscle tissues and serum of AF patients (Wang et al., 2019b). According to bioinformatics, the expression of LINC00472 in BC and CRC was possibly regulated by promoter methylation (Du et al., 2020; Fan et al., 2020). Moreover, the promoter region of LINC00472 in AF patients was also hyper-methylated. The high CpG methylation levels indicate a critical regulatory role in LINC00472 expression (Wang et al., 2019b).

#### Sepsis-Induced Acute Hepatic Injury

Sepsis, whose incidences are increasing annually, is a common disease in intensive care units (ICUs) with an incidence as high as 50% (Fan et al., 2020; Xu et al., 2021d). Sepsis, with the accompanying systemic inflammatory response syndrome, affects multiple systems and organs, culminating in multiple organ dysfunction syndrome and high mortality rates (Zhao et al., 2021a; Qiu et al., 2021). The liver is one of the main organs for endotoxin removal, therefore, patients with sepsis are vulnerable to liver injury (Xie et al., 2002; Cao et al., 2021). Currently, the mechanisms involved in acute hepatic injury (AHI) during sepsis are unknown. Expression levels of LINC00472 were found to be higher in rats with sepsis-induced AHI, relative to control group rats (Li et al., 2020a). Studies have also shown that LPS-treated models exhibit elevated ALT and AST levels. Besides, expressed of associated indicators have been shown to be suppressed in LPS + sh-LINC00472 administered groups (Li et al., 2020a). In addition, when LINC00472 was silenced, inflammatory cytokine levels, including IL-6, TNF- α and IL-10, were suppressed (Li et al., 2020a). These findings show that suppressed LINC00472 levels ameliorate sepsis-induced AHI progression.

#### Regulatory Roles of LINC00472 in Human Diseases

**FIGURE 3:**
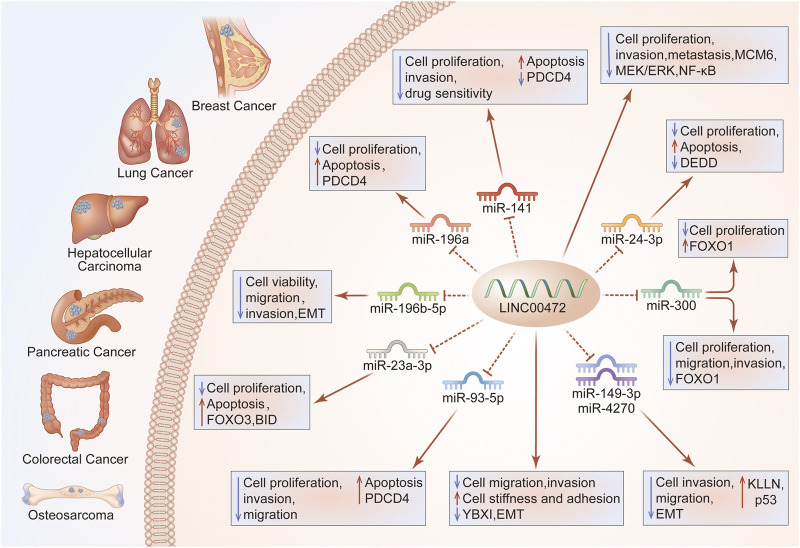
LINC00472 regulation of cell growth, and invasion. Through the ceRNA axis, LINC00472 regulates cancer cell growth and invasion. Regulation of the LINC00472/miR-141/PDCD4 pathway in BC enhances cell growth, invasion, drug sensitivity and apoptosis; LINC00472 inhibits BC cell proliferation, invasion, and metastasis by regulating the MCM6/MEK/ERK pathway; LINC00472 promotes DEDD expressions by sponging miR-24-3p, inhibiting LUAD cell proliferation and inducing apoptosis; Effects of LINC00472 on LC cell viability, migration, invasion, and EMT are attributed to direct targeting of miR-196b-5p; Overexpressed LINC00472 upregulates KLLN, inhibiting NSCLC cell proliferation, invasion, and EMT via the downregulation of miR-149-3p and miR-4270; LINC00472 inhibits EMT by binding to YBX1, which affects the mechanical properties of cells, ultimately inhibiting their abilities to invade and metastasize; The miR-93-5p/PDCD4 pathway mediates the suppressive functions of LINC00472 in HCC cells; LINC00472 promotes osteosarcoma tumorigenesis by suppressing FOXO1 expressions via miR-300; In CRC, LINC00472 inhibits proliferation and initiates apoptosis by increasing PDCD4 expressions through sponging miR-196a; In PC, silencing of LINC00472 suppressed the levels of BID via miR-23a-3p/FOXO3, which promoted PC cell proliferation while inhibiting their apoptosis.

**FIGURE 4:**
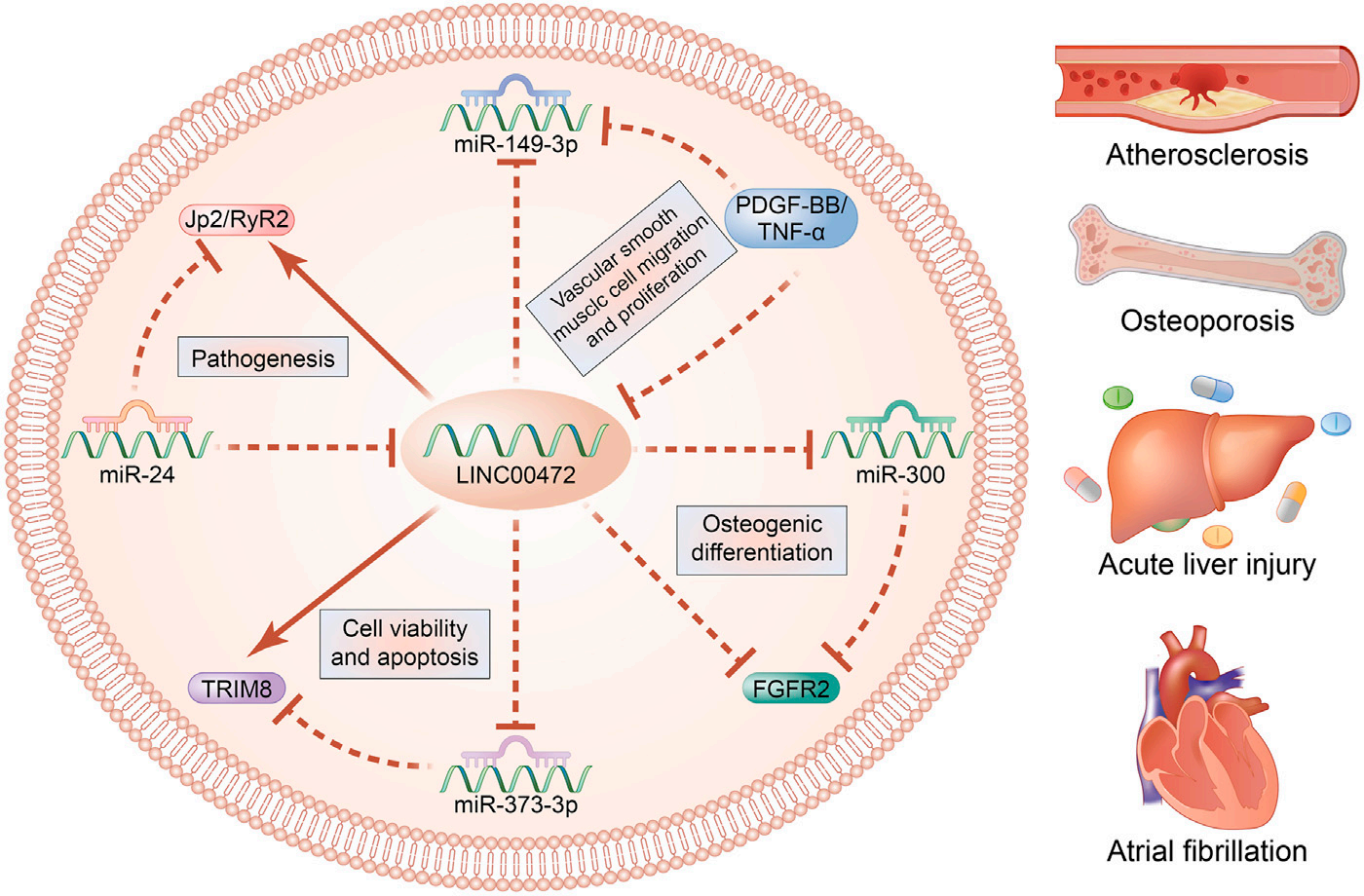
LINC00472 regulatory mechanisms in non-neoplasms.

LINC00472 is involved in several complex regulatory mechanisms and it plays a significant function in occurrence and development of multiple diseases. Regulatory mechanisms of LINC00472 are summarized in Figures 3, 4.

#### The MEK/ERK Signaling Pathway

In cancer cells, the MEK/ERK signaling pathway is highly activated (Gao et al., 2019; Yang et al., 2020a). This pathway is closely associated with tumorigenesis, including BC (Nyati et al., 2021), HCC (Chen et al., 2021b), OS (Liu et al., 2021f), CRC (Huang et al., 2021), and multiple myeloma (Zhou et al., 2021), where it regulates cell growth, apoptosis, invasion as well as metastasis. Based on its regulatory roles in cell survival in different cancer stages, the MEK/ERK pathway is a promising therapeutic target for cancer (Zheng et al., 2019). Functions of the MEK/ERK signaling pathway in BC have been reported (Qin et al., 2017; Wise and Zolkiewska, 2017). Nyati et al. documented that activated MEK/ERK signaling pathway significantly enhances breast cancer metastasis (Nyati et al., 2021). Overexpressed LINC00472 was shown to suppress the oncogenic properties of TNBC cells *in vitro* while suppressing tumor growth *in vivo* by down-regulating MCM6 and blocking the MEK/ERK signaling pathway (Shao et al., 2021), implying that MEK/ERK is a possible target of LINC00472.

#### NF-κB Signaling Pathway

Nuclear factor-kB (NF-κB), a transcription factor, plays vital roles in tumor initiation, progression, and tumorigenesis by directly modulating cell functions, including proliferation, survival, apoptosis inhibition, and metastasis as well as migration. It is composed of five members, p50 (RELB), p65 (RELA), c-REL, NF-κB2 and NF-κB1 (Ren et al., 2021b; Wang et al., 2021c; Espinosa and Marruecos, 2021). Activated NF-κB is correlated with phosphorylations of IκBα. IκBα degradation releases NF-κBs, which translocate into the nucleus to initiate oncogene transcription (Wang et al., 2021c). IκBα is a suppressor of the NF-κB pathway. Under specific conditions, IκB modifications affect NF-κB activities (Espinosa and Marruecos, 2021). Elevated constitutive NF-κB activities and suppressed IκBα levels have been reported in lung adenocarcinoma cells. IκBα phosphorylation as well as degradation by IKK to activate NF-κB signaling constitutes the canonical pathway (Ho et al., 2021). The p65 subunit has been found to have a transcription activation domain and it plays various roles in cell survival, proliferation, invasion, metastasis, tumor angiogenesis, as well as chemoresistance (Ren et al., 2021b). P65 is phosphorylated at multiple sites, and Ser536-phosphorylation enhances the transactivation potential of p65 (He et al., 2021). Functional regulation of p65 is highly correlated with its ubiquitination (Ren et al., 2021b). NF-κB has also been found to be constitutively active in BC, and its sustained activations are vital for BC progression and metastasis (Zhu et al., 2021b). However, lncRNAs and the NF-κB signal pathway have been documented in BC. Wang et al. documented that NF-κB phosphorylation can be suppressed by LINC00472 overexpression (Wang et al., 2019a). Overexpressed LINC00472 significantly suppresses IκBα as well as p65 phosphorylations, implying that NF-κB is a potential LINC00472 target (Wang et al., 2019a). Analyses of IκB kinase (IKK), which is involved in NF-κB activation, revealed that LINC00472 interacts with IKKβ (Wang et al., 2019a). Through cell experiments, it was verified that LINC00472 suppresses p65 and IκBα phosphorylations by binding IKKβ (Wang et al., 2019a). These studies suggest that LINC00472 may be a regulator of the NF-κB pathway.

#### The p53 Signaling Pathway

The p53 transcription factor, which is 16–20 kb in length, is composed of a pair of alleles and is a tumor inhibitor gene. It is localized on human chromosome 17 (Li et al., 2020b). The p53 tumor suppressor, known as the “genome guardian,” plays key roles in preventing tumor development (Li et al., 2020c). Not only is p53 a tumor inhibitor, it is also a transcription factor involved in modulation of various genes that play various roles in proliferation, cell-cycle arrest as well as apoptosis (Li et al., 2020b; Li et al., 2020c; Zhao et al., 2020). Mutations of p53 have been reported in over 50% of malignancies (Zhao et al., 2020). Moreover, dysregulated lncRNAs can affect p53 expression, thereby promoting tumor development and progression (Feng et al., 2020). Emerging evidence suggests that p53 can activate downstream targets to initiate cell cycle arrest and apoptosis. Moreover, several lncRNAs are involved in the p53 signaling network where they are p53 effectors or modulators (Li et al., 2020b; Chaleshi et al., 2020; Chen et al., 2020; Lu et al., 2020; Aravindhan et al., 2021; Xu et al., 2021e; Liu et al., 2021g; Lu et al., 2021; Wu et al., 2021). For instance, lncRNA GClnc1 contributes to ovarian cancer growth via p53 signaling pathway regulation (Li et al., 2020b). Overexpressed DDX11-AS1 arrests the binding of PARP1 to p53, which suppresses p53 levels in HCC (Xu et al., 2021e). Moreover, LINC00472 is a potential p53 signaling pathway regulator. Zou et al. documented that overexpressed LINC00472 activates the p53 pathway and suppresses NCI-H1299 cell EMT, migration, as well as invasion by upregulating KLLN levels in NSCLC (Zou et al., 2019).

#### MicroRNA-Mediated Regulation

LncRNAs are competing endogenous RNAs (ceRNAs) in various diseases (Sui et al., 2016). The ceRNA hypothesis implies a novel regulatory mechanism between coding and non-coding RNAs. Specifically, as molecular sponges, lncRNAs bind miRNAs through miRNA response elements (MREs) and subsequently suppress the expressions of mRNAs (Wang et al., 2018; Yang et al., 2020b). Aberrant levels of vital lncRNAs in the ceRNA network have significant effects on lncRNA/mRNA ceRNA crosstalks that are miRNA-regulated, thus contributing to cancer development as well as progression (Zhu et al., 2017). LncRNAs have been reported to regulate miRNA functions by acting as endogenous sponges to control the expressions of target genes. Moreover, miRNAs regulate the stability of lncRNAs by binding them (Ballantyne et al., 2016). A ceRNA network theory was proposed to discuss the associations among these three RNA transcript types. Currently, efforts are aimed establishing the mechanisms through which lncRNAs exert their biological roles in human diseases.

LINC00472 was shown to alter the proliferations, apoptosis, and invasions of breast cancer cell lines by modulating the miR-141/programmed cell death 4 (PDCD4) axis (Lu et al., 2018). Moreover, over-expressed LINC00472 elevated doxorubicin (ADR) sensitivity and promoted ADR-induced apoptosis in BC cell lines via the miR-141/PDCD4 axis (Lu et al., 2018). LINC00472 inhibits LC cell proliferations and increases their apoptosis by suppressing miR-24-3p and upregulating death effector domain-containing DNA-binding protein (DEDD) (Su et al., 2018). Overexpression of LINC00472 by miR-4270 or miR-149-3p may inhibit NSCLC cell proliferation (Zou et al., 2019). Moreover, up-regulated LINC00472 promotes LC cell migration, invasion, and apoptosis by inhibiting miR-196b-5p (Mao et al., 2019). Suppression of HCC cell growth, migration, as well as invasion by LINC00472 is mediated by the miR-93-5p/PDCD4 signal pathway (Chen et al., 2019). Furthermore, aberrant LINC00472 expressions increased the abundance of HCC cells in the G1 phase and decreased cell counts in S phase via the miR-93-5p/PDCD4 signaling pathway (Chen et al., 2019). Zhang et al. (2020) reported that LINC00472 expression inhibits cell growth, migration, as well as invasion via regulation of the miR-300/FOXO1 signaling pathway in OS cells. Interestingly, in colorectal cancer, upregulated miR-196a reversed LINC00472-mediated cell growth and PDCD4 silencing reversed cell proliferation as well as apoptosis (Ye et al., 2018). In the pancreatic cancer cell line (BXPC3), western blot and RT-qPCR assays showed that BID levels were inhibited, while expression levels of miR-23a-3p were elevated after individual silencing (Bi and Wang, 2021). Elevated miR-23a-3p in pancreatic cancer exhibited negative correlations with LINC00472 levels (Bi and Wang, 2021). The RNA pull-down assay showed that binding between LINC00472 and miR-23a-3p was promoted after LINC00472 overexpression, while binding between FOXO3 and miR-23a-3p was alleviated (Bi and Wang, 2021). LINC00472 knockdown enhances pancreatic cancer cell proliferation and impedes their apoptosis by suppressing FOXO3 (Bi and Wang, 2021). FOXO3 transcriptionally activates BID expression (Bi and Wang, 2021). LINC00472 knockdown inhibits BID expressions via miR-23a-3p/FOXO3 to regulate the proliferation as well as apoptosis of pancreatic cancer cells (Bi and Wang, 2021).

In non-neoplastic diseases, HCM cells and H9C2 cells that had been transfected with pcDNA-LINC00472 or si-miR-24 exhibited suppressed miR-24 levels and elevated JP2 mRNA/protein and RyR2 protein levels, implying the presence of negative relationships among miR-24, JP2, LINC00472 and RyR2 in regulation of AF (Wang et al., 2019b). Besides, a novelty mechanism is suggested that LINC00472 affects bone differentiation by sponging miR-300 to regulate FGFR2 expression (Guo et al., 2020). Negative regulation of LINC00472 and TRIM8 as well as positive regulation of miR-373-3p improves cell viability and accelerates cell apoptosis, thereby alleviating sepsis-induced AHI (Li et al., 2020a). Additionally, upregulated LINC00472 increases the migration rate of VSMCs in atherosclerosis by regulating miR-149-3p (Jing et al., 2021).

Chemoresistance is a leading cause of therapeutic failure in cancer (Xu et al., 2021d). Therefore, understanding the underlying mechanisms of chemotherapy resistance is critical for improving chemotherapy (Zhao et al., 2021a). Over-expression of LINC00472 enhanced doxorubicin (ADR) sensitivity and promoted ADR-mediated apoptosis in BC cell lines via the miR-141/PDCD4 axis (Lu et al., 2018).

### Clinical Applications of LINC00472 in Human Diseases

Studies have evaluated the diagnostic, treatment, and prognosis-related biological targets of lncRNAs in various diseases, particularly cancers (Lv et al., 2019; Zhang et al., 2019; El-Ashmawy et al., 2021). Even though substantive advances have been made in tumor therapy, efficacious therapies for solid tumors are yet to be developed (Xue et al., 2021). Currently, studies on LINC00472 are in their early stages, and LINC00472 levels in human diseases have not been fully reported. Moreover, functional roles as well as relationships of LINC00472 and diseases have not been conclusively determined. Thus, LINC00472 is a potential marker for the diagnosis, metastasis, prognosis and prediction of clinical therapeutic responses in solid tumors. Although reports on LINC00472 are limited in non-tumor disease, the current evidence suggests that LINC00472 is a potential marker and a promising target for cancer diagnosis/prognosis and therapy.

#### LINC00472 and Diagnosis/Prognosis

The ability of lncRNAs to predict disease prognosis has been proven in several studies (Kumar et al., 2016; Zhou et al., 2018; Zhao et al., 2021b). Patients with AF were found to exhibit reduced plasma LINC00472 levels. Further assessment of receiver operating characteristic (ROC) curves revealed that the AUC value of LINC00472 was 0.86 (Wang et al., 2019b), implying that LINC00472 has a significant diagnostic potential in AF.

In 2021, the most clinically relevant finding was that Log10 LINC00472 expression was correlated with PBC. These findings confirmed that LINC00472 is not only highly expressed in cholangitis patients, but is also higher in advanced patients than in early stage patients and healthy people (Dong et al., 2021). However, the positive correlation between serum collagen type IV (C-IV) levels and relative expressions of log10 LINC00472 was intriguing, but not surprising (Dong et al., 2021). Thus, LINC00472 has the potential to be a novel biomarker for the diagnosis of PBC.

In human BC patients, high expressions of LINC00472 were positively correlated with overall survival (*p* = 0.005) and disease-free (*p* < 0.001) outcomes (Shen et al., 2015a). Kaplan-Meier survival analysis showed that suppressed LINC00472 levels are associated with poor overall survival, relative to elevated LINC00472 levels (*p* = 0.0014, log-rank test) (Lu et al., 2018). In addition, overall survival rates were markedly low in HCC patients with suppressed LINC00472 levels, relative to patients with elevated LINC00472 levels (*p* = 0.0045). These findings imply poor prognostic outcomes for patients with suppressed LINC00472 levels (Chen et al., 2019). Zou et al. (2019) reported that elevated LINC00472 levels are correlated with better survival outcomes in NSCLC patients. Therefore, LINC00472 may be a valuable prognostic target in various diseases.

#### LINC00472 and Therapy

LINC00472 is a potential treatment target for various diseases. The first study to draw the attention of scientists to LINC00472 was published in 2015 (Shen et al., 2015a). According to this study, LINC00472 is highly abundant in BC patients with positive hormone receptors than in those with negative receptor status (*p* < 0.0001), and it is correlated with tumor grade (*p* < 0.0001), tumor sizes (*p* < 0.0001), as well as disease stage (*p* = 0.007) (Shen et al., 2015a). Notably, patients with overexpressed LINC00472 were established to be more sensitive to assisted chemotherapy (*p* = 0.021) or hormonal therapy (*p* = 0.003) than patients with suppressed LINC00472 (Shen et al., 2015a). Concurrently, overexpressed LINC00472 is strongly associated with the risk of relapse and death (*p* = 0.043) (Shen et al., 2015a). Besides, LINC00472 expression levels have been positively correlated with the progression of lymph-node metastasis and distant metastasis in BC (Lu et al., 2018). Furthermore, the role of LINC00472 in drug sensitivity has been reported in BC (Lu et al., 2018). LINC00472 expressions were established to be considerably suppressed in triple-negative breast cancer (TNBC) tissues, with levels correlating with lymph node metastasis, histological grading and clinical grading (*p* < 0.05) (Shao et al., 2021). Tumor tissue samples from LUAD and NSCLC patients exhibited low levels of LINC00472 (Su et al., 2018; Zou et al., 2019). In addition, aberrantly expressed LINC00472 in LUAD and NSCLC patients are strongly associated with TNM clinical staging (Zou et al., 2019; Deng et al., 2020). As previously reported, LINC00472 levels are suppressed in HCC tissues, compared to adjacent normal tissues (Chen et al., 2019). Notably, LINC00472 levels were found to be downregulated in HCC metastatic tissues, relative to non-metastatic tissues (Chen et al., 2019). LINC00472 expressions in epithelial OC have been associated with disease stage and tumor grade (Fu et al., 2016). Corresponding numbers were 45.21 versus 28.04% when comparing disease stage (stage I or II disease versus stage III or IV) (*p* = 0.024), and 51.72 versus 30.47% when comparing tumor grade (grade 1 tumors versus grade 2 or three tumors) (*p* = 0.004) (Fu et al., 2016). In pancreatic cancer, by evaluating the relationship between LINC00472 levels and clinic-pathological characteristics, Bi et al. (Bi and Wang, 2021) established obvious associations between LINC00472 levels and patient’s TNM stages as well as lymph node metastasis (*p* < 0.05). Moreover, patients with low expression levels were associated with inferior overall survival outcomes. LINC00472 levels were found to be low in both cardiac muscle tissues and serum of AF patients (Wang et al., 2019b). In a recent study, LINC00472 levels were significantly low in osteoporosis patients, compared to healthy control individuals (Guo et al., 2020). LINC00472 has also been studied in atherosclerotic coronary tissues, where it was found to be highly expressed (Jing et al., 2021).

Therefore, LINC00472 is a potential therapeutic target for several diseases, and it influences disease progression by regulating LINC00472/miRs or molecular pathways. Investigations of biological mechanisms have shown that LINC00472 is a potential biomarker for therapeutic outcomes.

## Conclusions and Future Perspectives

LINC00472 is involved in the development of various diseases, making it a potential marker for disease diagnosis, treatment, and prognosis, particularly in tumors. In this review, we highlighted the functions and mechanisms of LINC00472 in various diseases. Aberrantly expressed LINC00472 is a promising approach to disease treatment and prognosis. Plasma LINC00472 levels are ideal disease diagnostic factors. However, identification of diagnostic biomarkers for diseases other than AF and PBC remains difficult. Even though studies have evaluated the relationship between LINC00472 and disease characteristics, such as disease stage, tumor grade, disease-free, overall survival, lymph node metastasis, and TNM stage, their roles as therapeutic targets for diseases are yet to be thoroughly investigated. Moreover, studies have mostly focused on regulatory mechanisms of LINC00472 in various diseases. Aberrantly expressed LINC00472 influences cell proliferation, migration, invasion, as well as apoptosis. Moreover, it regulates various signaling pathways, including MEK/ERK, NF-κB, and p53 signaling pathways. Meanwhile, LINC00472 has also been established to be a tumor suppressor in BC, LC, HCC, OS, CRC, OC, PC, and GC, particularly during carcinogenesis and cancer progression. LINC00472 acts as a competing endogenous RNA (ceRNA) to sponge miRNAs, such as miR-141, miR-24-3p, miR-196b-5p, miR-149-3p, miR-4270, miR-93-5p, miR-300, and miR-196a. However, there is a need to elucidate on the significance of LINC00472-miRNAs in diseases. The relationship between LINC00472 and DNA methylation has also been investigated. In conclusion, LINC00472 is a potential treatment target for several diseases.
